# Effect of High Calcium Fly Ash, Ladle Furnace Slag, and Limestone Filler on Packing Density, Consistency, and Strength of Cement Pastes

**DOI:** 10.3390/ma14020301

**Published:** 2021-01-08

**Authors:** Eleftherios K. Anastasiou

**Affiliations:** Department of Civil Engineering, Aristotle University of Thessaloniki, 541 24 Thessaloniki, Greece; elan@civil.auth.gr

**Keywords:** supplementary cementitious materials, packing density, filler effect, high calcium fly ash, ladle furnace slag, limestone filler

## Abstract

Environmental considerations and technical benefits have directed research towards reducing cement clinker content in concrete, and one of the best ways to do this is to replace cement with supplementary cementitious materials. High calcium fly ash, ladle furnace slag, and limestone filler were investigated as supplementary cementitious materials in cement pastes, and binary mixtures were produced at 10%, 20%, and 30% cement replacement rates for each material. The water requirement for maximum packing and for normal consistency were obtained for each paste, and strength development was determined at 3, 7, 28, and 90 days for the 20% replacement rate. Furthermore, two ternary mixtures at 30% cement replacement were also prepared for maximum packing density and tested for compressive strength development. The results showed that high calcium fly ash decreased cement paste packing and increased water demand but contributed to strength development through reactivity. Ladle furnace slag and limestone filler, on the other hand, were less reactive and seemed to contribute to strength development through the filler effect. The ternary paste with 70% cement, 20% high calcium fly ash, and 10% limestone filler showed equivalent strength development to that of the reference cement paste.

## 1. Introduction

Supplementary cementitious materials (SCMs) are used increasingly in cement-based products, either for improving their properties or for reducing the carbon footprint of cement. Given that the hydration of ordinary Portland cement (OPC) is not yet understood in full, these materials bring even more complex reactions into the hydration process [1,2]. The benefit from utilization of SCMs lies either in their reactivity [3,4] or in the enhancement of cement hydration, as explained by the filler effect [5,6]. The environmental benefit from cement replacement with SCMs increases with the rate of replacement [7,8], but cement substitution must be limited to the extent that the performance of the final product is not undermined. The effect of SCM use could be beneficial to the performance of the produced concrete, depending on its fineness and on its cement substitution rate [9,10]. Cement substitution by a SCM is expected to affect the rate of strength development and the final strength, but also the water requirement and consistency of the cement paste [11,12].

SCM particles have a different size and specific surface area compared to Portland cement and, therefore, alter the microstructure and packing density of the cement paste. The particle size distribution of the cementitious materials has been found to influence both workability and hydration of cement pastes by improving their packing density [13,14]. Mixture design optimization by considering particle packing has been utilized in the design of ultra-high performance concrete [15,16]. There have been several mathematical packing models proposed to predict the packing density of multi-component mixes [17,18]. In order to predict the effect of cement substitution with SCMs, Yu et al. [19] proposed a linear packing model, considering the surface characteristics (sphericity) of particles. De Larrard [20] proposed the compressive packing model, considering the degree of compaction rather than particle surface characteristics, while Fennis et al. [21] proposed the compaction-interaction packing model, considering particle interaction and aggregate inclusion. According to Zhang et al. [22], the fresh cement paste can be seen as a suspension, with water either filling the voids between particles (filling water) or coating particles and providing fluidity (excess water). SCMs can act as fillers, improving the packing density of the suspension, which means that they can reduce the required amount of filling water. They can also provide space for the hydration of cement, accelerating strength development (filler effect). On the other hand, large SCM particles could block smaller cement particles from hydrating (wall effect) and a large proportion of small SCM particles could increase the distance between hydrated cement particles (loosening effect), affecting the consistency of cement pastes [23]. Mehdipour and Khayat [24] suggested that the presence of more fine particles than the amount required to fill the voids in the cement matrix contributes to the flowability of concrete. SCM particles themselves, on the other hand, may be contributing directly to strength development, if they exhibit hydraulic or pozzolanic properties. 

There are several well-known SCMs, such as siliceous fly ash, silica fume, ground granulated blastfurnace slag, and limestone filler (LF). Their availability, however, varies locally, and several other materials are being researched, based on local availability, such as high calcium fly ash (HCFA), metakaolin, ladle furnace slag (LFS), and rice husk ash [25]. The present research investigates the use of HCFA, a by-product of lignite-fired power plants; LFS, a by-product of the steelmaking process; and LF, ground natural limestone, in cement pastes. HCFA is known to exhibit both pozzolanic and self-cementing properties and has been used for the past decades in blended cement manufacturing [26,27]. LFS is a weak pozzolan with some latent hydraulic properties and is mostly considered as filler [28,29]. LF is receiving increasing attention in the literature as a SCM since it seems to promote cement hydration [30,31,32]. A combination of SCMs in ternary systems is often proposed since there seems to be some synergy between alternative materials of different chemical composition and of different fineness [33,34]. 

The aim of the present research was to investigate the effect of cement replacement with the above SCMs, considering packing density, in binary and ternary mixtures. Since the fineness and reactivity of the SCMs have an impact on fresh paste consistency and strength development, it is important to understand how increasing cement replacement and altering packing density affects these properties. Furthermore, the ternary binders consisting of OPC, HCFA and LFS or OPC, HCFA, and LF were studied to identify possible synergistic effects. The goal was to identify ways of designing cement pastes with SCMs in the most beneficial way possible, since successful implementation can result in maximizing the positive effects of cement substitution and increasing SCM use.

## 2. Materials and Methods 

CEM type I 42.5 N according to EN 197-1 [35] was used as OPC for all the tests. HCFA was used unprocessed, as received from the power plant. LFS was water-quenched and air-cooled and then sieved through the 100 μm sieve. LF was used as received from the cleaning of the limestone aggregate silos in a ready-mixed concrete plant. Table 1 shows the chemical composition of all the materials used, measured by atomic absorption spectroscopy (AAnalyst 400, Perkin Elmer, Waltham, MA, USA). The loss on ignition (LOI) and the chloride, sodium, and sulfate ion contents were determined by ionic chromatography (Thermo Scientific, Waltham, MA, USA, Dionex ICS-1100) for all the materials used. 

**Figure 1:**
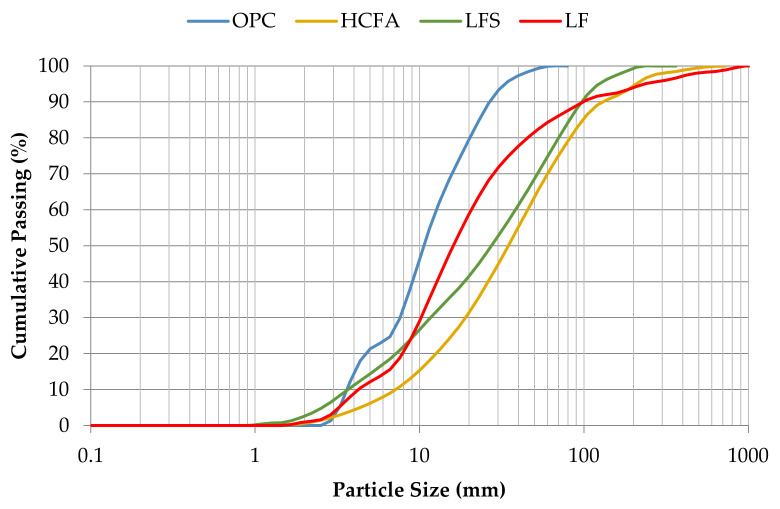
Particle size distribution of the fine materials used.

Figure 1 shows the particle size distribution of the materials used, and Table 2 shows their median particle size diameter d50, specific surface area, and apparent specific density values. The particle size distribution, d50, and specific surface area of the fine materials were measured using a laser particle size analyzer (Malvern Mastersizer 2000, Worcestershire, United Kingdom). The apparent specific density of the fine materials was determined using a Le Chatelier flask, according to ASTM C188-14 [36].

The first step in the experimental program was to identify water demand when substituting OPC with each of the three alternative binders. The required water to cementitious material (w/cm) ratios were determined both for maximum packing density and for equal consistency. The wet packing density approach, as proposed by Wong and Kwan [36], was followed in order to determine packing density for pastes with 100% OPC and for pastes with 10%, 20%, and 30% cement replacement with HCFA, LFS, and LF. The w/cm for maximum packing was recorded, referred to as optimum water demand. The reduction of the w/cm ratio increases packing density up to the point that the water fills the voids amongst solid particles, but further water reduction decreases packing. Thus, the optimum water demand is determined at the point where packing density is maximized. However, at maximum packing, the workability of the fresh paste is typically very low and serves as a measure of the effect of SCM use on packing and maximum strength development. In order to determine water demand for workable pastes, the w/cm ratio for normal consistency, according to the Vicat method as described in European Standard EN 196-3 [37], was also determined for the same replacement rates.

The cement pastes were prepared in a laboratory mixer by adding water first and then adding the dry-mixed binders and mixing for 120 s. Additional mixing time of 30 s was allowed if required. The fresh pastes were placed in the 40 mm deep truncated conical Vicat mold and compacted on a vibration table for elimination of entrapped air and weighed for the determination of wet packing. Wong and Kwan [38] have identified packing density as the solid concentration *φ*, which is calculated from Equations (1) and (2) as follows:

(1) $$\varphi =\frac{V_{c}}{V}$$

where *V*_c_ is the solid volume of the cementitious materials and *V* is the volume of the mold. The solid volume *V*_c_ can be calculated from the following formula:

(2) $$V_{c}=\frac{M}{\rho_{w}u_{w}+\rho_{\alpha }R_{\alpha }+\rho_{\beta }R_{\beta }}$$

where *M* is the mass of the paste in the mold; *ρ_w_*, *ρ_α_*, *ρ_β_* are the densities of water and cementitious materials *α* and *β*, respectively; *u_w_*is the water to cementitious material ratio by volume (w/cm_V_); and *R_α_* and *R_β_* are the volumetric ratios of cementitious materials *α* and *β*, respectively.

After weighing, the truncated conical specimens, still in the mold, were subjected to measurement of Vicat plunger penetration, according to EN 196-3, and the depth of penetration was recorded. The depth of plunger penetration, with a minimum of 0 mm and a maximum of 40 mm, characterizes the consistency of the paste and was used as a measure of workability. Normal consistency is described in the standard as the consistency that allows the plunger to penetrate the specimen 34 mm. Lecomte et al. [39] followed a similar approach to characterize the packing ability of various cement pastes. A series of pastes, 8 to 12 for each paste formulation, was prepared for various w/cm ratios in order to identify the optimum water demand for maximum packing and to determine the w/cm ratio for a paste of normal consistency, which was selected as a suitable level of workability. The above procedure was carried out for 100% OPC as reference and for 10%, 20%, and 30% wt. OPC replacement with HCFA, LFS, and LF, resulting in ten different formulations.

Based on the w/cm ratios for optimum water demand and for normal consistency, binary pastes with 20% OPC replacement with HCFA, LFS, or LF were prepared and tested for compressive strength development at 3, 7, 28, and 90 days. At least six 40 mm cubic specimens were tested at each age and paste, after curing at 20 °C and 95% relative humidity. These were compared to reference 100% OPC cement pastes and were used to assess the contribution of each SCM to strength development, either at maximum packing (optimum water demand), or at equal fresh state workability (normal consistency). Since the tested SCMs had varying effects on workability and strength development, it was decided to test ternary binders to identify possible benefits from the interaction of binders. Based on the strength development test results, two ternary cement pastes, one with 70% OPC, 20% HCFA, and 10% LFS and one with 70% OPC, 20% HCFA, and 10% LF were prepared and tested for strength development at 3, 7, 28, and 90 days at optimum water demand. Scanning Electron Microscopy (SEM-JEOL 840A JSM, Tokyo, Japan) was also used to assess the crystals formation and the microstructure of the reference and ternary cement pastes.

## 3. Results

### 3.1. Material Properties

The chemical composition of the materials, as shown in Table 1, shows that all the binders used were rich in calcium. This is expected to influence the kinetics of hydration in a different way compared to traditional silica-rich SCMs (siliceous fly ash, silica fume, ground granulated blastfurnace slag), especially due to the presence of free-CaO in HCFA and LFS; HCFA has both hydraulic and pozzolanic properties, while LFS can be described as a weak pozzolan with latent hydraulic properties and is often regarded as filler [40,41]. HCFA shows a relatively high sulphate ion content, which is not expected to affect cement hydration negatively [41].

Although SCM use for increasing particle density often relies on materials finer than cement, the particle size distribution of the SCMs used shows that all of them were coarser than cement. According to the literature, however, coarser SCMs may still contribute to cement hydration through the filler effect [13,22,42]. HCFA is the coarsest material, considering both median particle size diameter d50 and specific surface area. LFS and LF have similar specific surface areas but different particle size diameters. This occurrence can be explained by their surface characteristics, as LF is ground natural stone, resulting in more spherical shaped particles compared to LFS, which is molten and then water-quenched, resulting in more irregularly shaped particles. According to Sakai et al. [43], spherical-shaped particles are expected to increase packing density and fluidity of cement-bound mixtures.

### 3.2. Effect on Wet Packing Density

**Figure 2:**
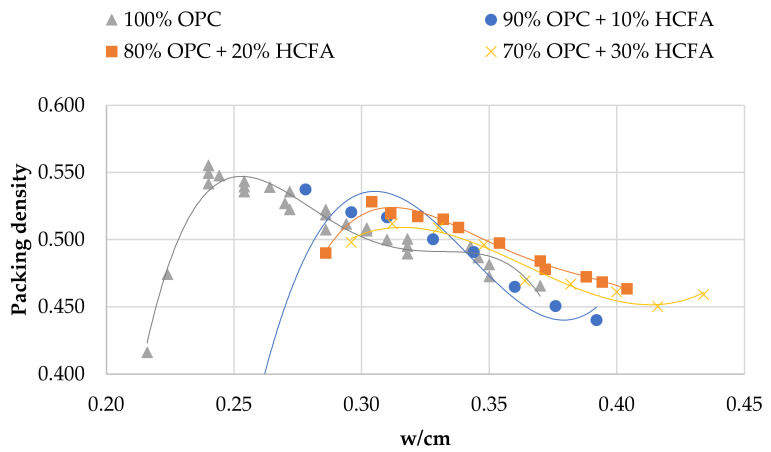
Packing density vs. w/cm in pastes with OPC and HCFA.

**Figure 3:**
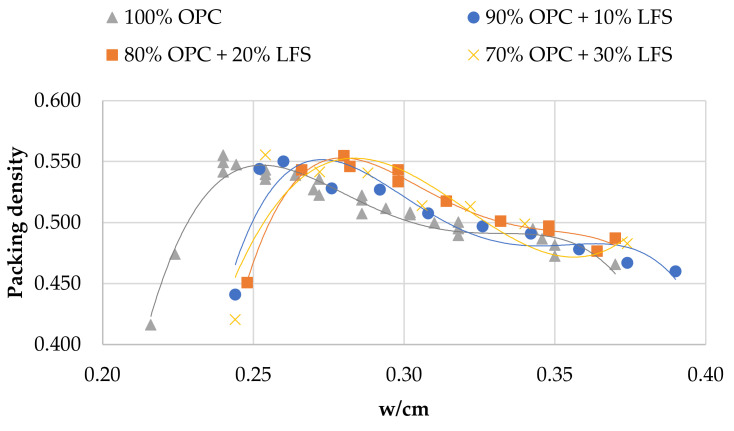
Packing density vs. w/cm in pastes with OPC and LFS.

**Figure 4:**
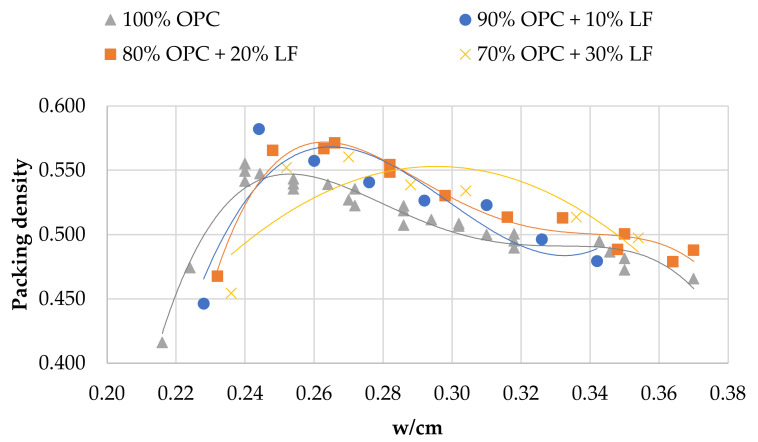
Packing density vs. w/cm in pastes with OPC and LF.

Figure 2, Figure 3 and Figure 4 show the relationship between packing density and w/cm ratio for various cement replacement rates with SCM. As can be seen in all cases, reducing w/cm ratio increases packing up to the point where the water is not sufficient to fill the voids between the particles. From the curves of the figures, it is easy to estimate the w/cm ratio for the maximum packing, but it is also possible to estimate the effect of each SCM on water demand and its impact on packing density of the cement paste. The use of HCFA, as shown in Figure 2, increases water demand considerably, even at 10% OPC replacement. It also reduces the solid concentration in the paste in all cases.

Cement replacement with LFS, on the other hand, seems to have a different effect. Although water demand was increased (to a lesser extent compared to HCFA), particle packing was equal or even slightly increased compared to that of the reference paste. As with HCFA, the rate of replacement, ranging from 10% to 30%, had little effect. Cement replacement with LF had a similar effect on packing to that of LFS, but more pronounced. At 10% and 20% OPC replacement rates with LF, packing density increased, while it decreased at the 30% replacement rate. Water demand increased, but only slightly.

The effect of cement replacement with the various SCMs on packing density seems in all cases to be linked with their fineness. Indeed, an analysis of variance shows that the influence of fineness on packing density is statistically significant (*p* < 0.05) for all the materials. The finer materials (LFS and LF) contribute to the increase of solid concentration, while the coarser material (HCFA) decreases packing. OPC substitution with 10% and 20% LF results in the highest packing densities, while the lowest are observed with 30% HCFA substitution. HCFA, and LFS to a lesser extent, require increased w/cm ratios in order to reach maximum packing. An analysis of variance shows that the influence of w/cm ratio on packing density is statistically significant (*p* < 0.05), which means that increased water demand, mostly for HCFA, but also for LFS, results in lower packing density. This increase in water demand, however, does not seem to be linked with fineness, but can be associated with chemical composition, and more specifically, with free lime content [44]. LF shows a slight increase in water demand, which can be attributed to the water absorption of limestone particles.

### 3.3. Effect on Consistency

**Figure 5:**
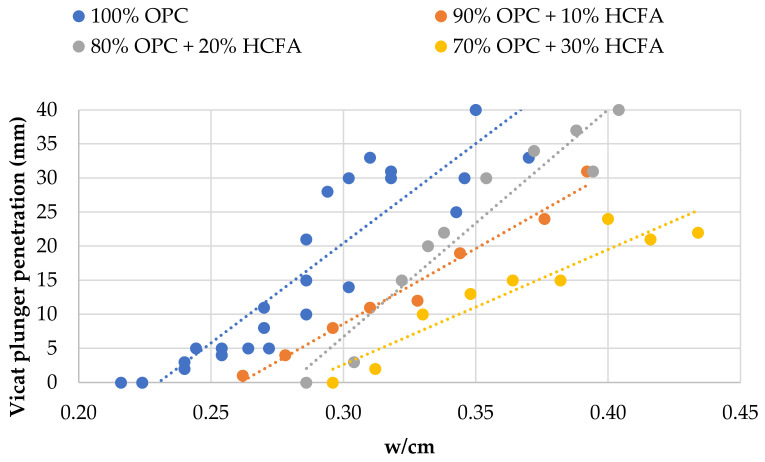
Effect of OPC replacement with HCFA on fresh paste consistency.

**Figure 6:**
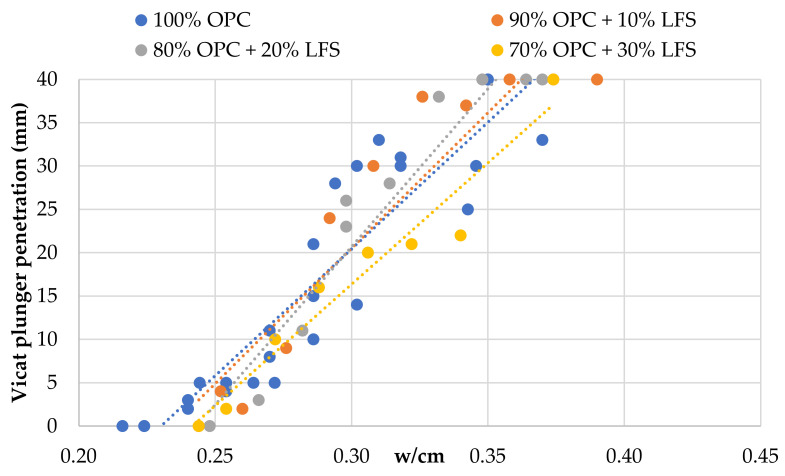
Effect of OPC replacement with LFS on fresh paste consistency.

**Figure 7:**
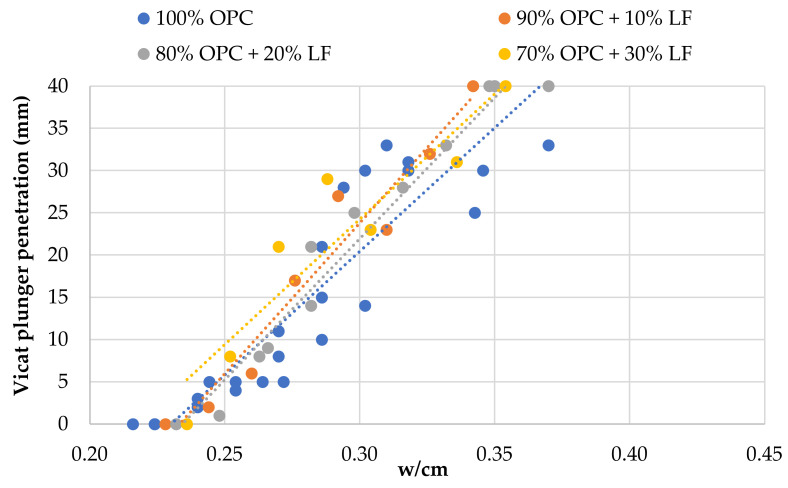
Effect of OPC replacement with LF on fresh paste consistency.

Figure 5, Figure 6 and Figure 7 show the relationship between w/cm ratio and consistency, as expressed by the Vicat plunger penetration, for each SCM. Figure 5 shows that OPC replacement with HCFA increases the w/cm ratio for normal consistency, and the w/cm ratio needs to be increased further for higher replacement rates. This can be explained due to the higher water demand of HCFA but also due to its negative effect on packing density. OPC substitution with LFS (Figure 6), on the other hand, seems to have a minimal effect on consistency, and the w/cm ratio for normal consistency remains unchanged regardless of the rate of cement replacement. The same seems to be the case for OPC substitution with LF (Figure 7), where there seems to be a small increase in consistency at the 30% replacement rate. Again, the effect of LFS and LF on the consistency of fresh cement pastes can be linked with their effect on packing density, which agrees with the literature [45,46].

### 3.4. Effect on Strength Development in Binary and Ternary Pastes

Table 3 shows the cement pastes tested for compressive strength development. The reference and binary mixtures were prepared either with w/cm ratios for maximum packing (optimum water demand) obtained from Figure 2, Figure 3 and Figure 4 or with w/cm ratios for standard consistence, obtained from Figure 5, Figure 6 and Figure 7. Two ternary mixtures were prepared with w/cm for maximum packing, determined following the same procedure as for the binary mixtures. 

**Figure 8:**
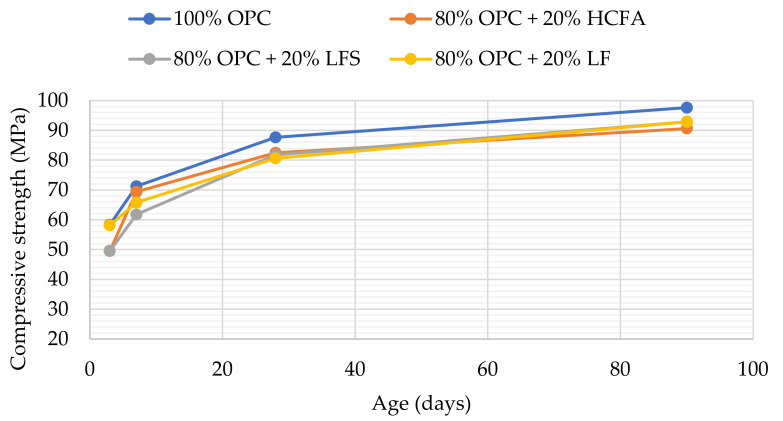
Compressive strength development at optimum water demand for binary pastes.

**Figure 9:**
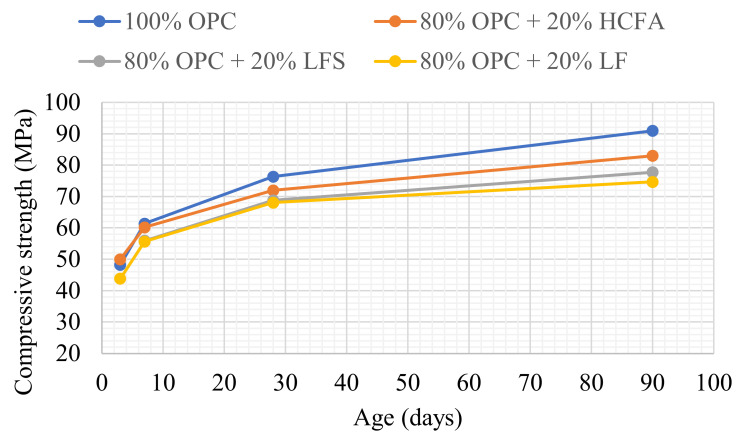
Compressive strength development at normal consistence for binary pastes.

Table 3 shows that 20% replacement with either HCFA, LFS, or LF at optimum water demand gave 92–94% of the 28-day compressive strength of the reference paste, despite lower packing density and increased w/cm. Figure 8 shows that the same pattern continued at 90 days, while HCFA had some accelerating effect at 7 days, which may be attributed to the presence of free lime. Strength development was similar for all of the SCMs in the binary systems, despite the fact that pastes with HCFA had higher w/cm ratios and reduced packing densities. For normal consistence, as shown in Figure 9, 20% OPC replacement with HCFA showed slightly better strength development compared to LFS and LF, again despite higher w/cm ratios and lower packing densities. The results show that the reactivity of HCFA had a greater effect on strength development than fineness, water demand, or packing. LFS and LF, on the other hand, seemed to be contribute to strength development mostly through the filler effect. 

**Figure 10:**
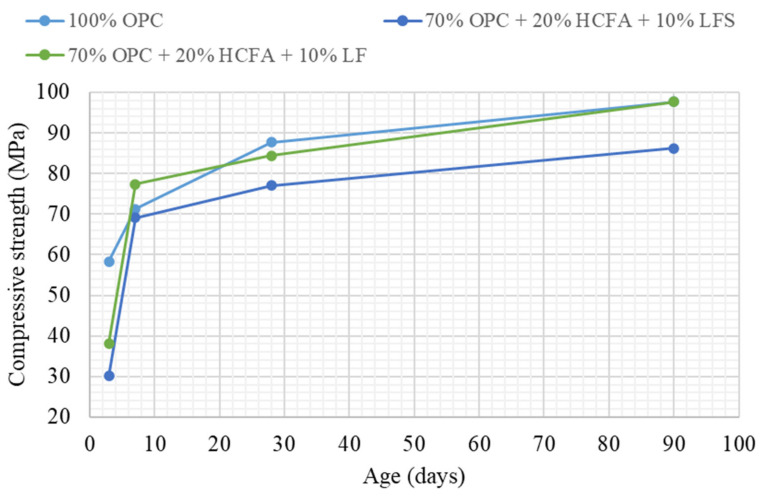
Compressive strength development at optimum water demand for ternary pastes.

Based on these results, it was decided to test ternary binders with 20% HCFA and 10% LFS or LF cement substitution. The results shown in Table 3 showed that the ternary binders reached a packing density close to that of the reference paste at a higher w/cm ratio; 28-day compressive strength, however, was higher when LF rather than LFS was used as the third constituent. The rate of strength development, as shown in Figure 10, shows that the accelerating effect at 7 days observed with 20% HCFA use was enhanced with an extra 10% LF replacement, while the 90-day compressive strength was equal to that of the reference.

The results show a good synergy between HCFA and LF, also identified by other researchers. De Weerdt et al. [47] suggest that limestone interacts with the hydration products of OPC–fly ash systems and increases compressive strength. Thongsanitgarn et al. [48] have shown that the increase in strength development of OPC–fly ash systems is higher when finer limestone is added. Other researchers point out that different SCMs may co-operate in the cement paste matrix, despite showing different chemical activity and physical characteristics [49]. The same synergy, however, did not take place when HCFA was combined with LFS, resulting in reduced strength development (88% of the reference at 28 and 90 days). A possible explanation for this is that the calcium in LF was in the form of CaCO_3_, which is known to promote cement hydration [50], while in LFS, it was mostly in the form of CaO or Ca(OH)_2_, as shown from the values of loss on ignition in Table 1.

**Figure 11:**
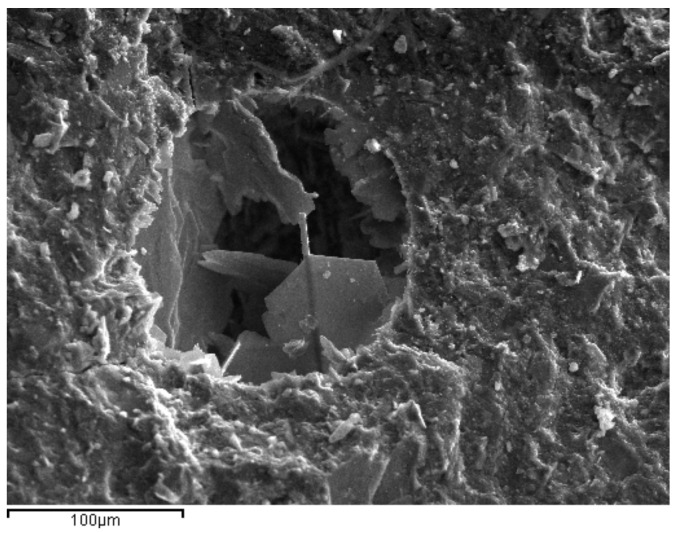
Portlandite crystal into a pore of 100% OPC paste at 28 days.

**Figure 12:**
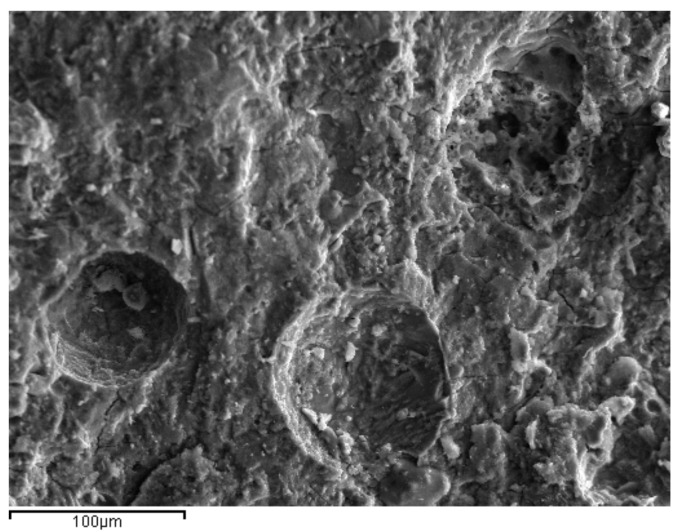
Microstructure of 70% OPC + 20% HCFA + 10% LFS paste at 28 days.

**Figure 13:**
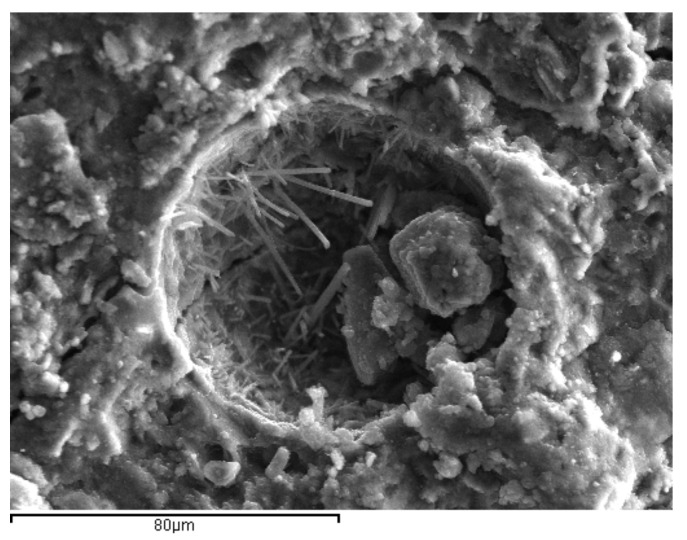
Effect of OPC replacement with LF on fresh paste consistency.

SEM photos taken from samples at 28 days were used to explore the microstructure of the reference and ternary pastes (Figure 11, Figure 12 and Figure 13). The pores observed were of similar size, while micro cracks were visible in all the cement pastes. Portlandite (Ca(OH)_2_) crystals were identified in the pores of the reference paste, as a result of cement hydration (Figure 11). Ettringite needle-shaped crystals, on the other hand, were visible in the pores of the ternary paste with 70% OPC + 20% HCFA + 10% LF, confirming enhanced reactivity in the pore solution.

## 4. Discussion

The results showed that the design of binders with SCMs can be optimized by considering their physical and mechanical characteristics. HCFA had reduced fineness and increased water demand compared to LFS and LF, which rendered pastes with OPC substituted with HCFA less workable. Combining OPC with HCFA and LFS or LF in ternary pastes showed a solution to this problem. Furthermore, considering packing density can aid the design process and help design pastes with either the lowest w/cm ratio at maximum solid concentration for strength optimization or with the w/cm ratio for the required consistency. Fineness has been shown to influence the packing density and consistency of pastes greatly, but reactivity and synergy between binders also seems to be very important for compressive strength development. The reactivity of HCFA seemed to contribute more to strength development compared to the finer LFS and LF, despite the increased water demand. Regarding fineness, the use of SCMs coarser than cement can still lead to equal packing densities but grinding the SCMs to higher fineness could lead to further improvements. The ternary mixtures with OPC, HCFA, and LFS or LF, however, showed packing densities similar to that of 100% OPC paste. HCFA with LF in ternary pastes with OPC showed good synergy in cement-based binders, reaching the strength development of the reference paste, despite having a higher w/cm ratio at the 30% replacement rate, which was not the case when LFS was used as the third constituent. Overall, it seems that designing ternary binders with suitable SCMs by considering particle packing could compensate for strength loss and result in equal performance with reduced cement content.

## Figures and Tables

**Table 1:** Chemical properties of the fine materials used in the present research (%wt.).

Constituents	OPC	HCFA	LFS	LF
CaO	66.80	47.20	50.70	51.30
SiO_2_	19.60	33.10	32.40	3.80
Al_2_O_3_	3.74	7.29	1.36	1.00
Fe_2_O_3_	2.40	4.00	2.66	0.40
MgO	3.91	3.20	2.77	1.20
Na_2_O	0.57	1.00	0.78	-
K_2_O	1.08	0.53	0.06	-
LOI	1.91	3.75	6.72	41.00
Cl^−^	0.03	0.04	0.02	-
NO_3_^−^	0.02	-	-	-
SO_4_^2−^	1.49	4.81	0.43	-

**Table 2:** Physical properties of the materials used.

Material	Median Particle Size Diameter d50 (μm)	Specific Surface Area (m^2^/kg)	Apparent Specific Density (kg/m^3^)
OPC	12.22	642	3140
HCFA	39.92	307	2420
LFS	31.47	496	2590
LF	18.45	480	2620

**Table 3:** Fresh and hardened paste properties.

Solid Constituents (% wt.)	w/cm Ratio	Packing Density	Consistency	28-Day Compressive Strength (MPa)
100% OPC	0.24	0.542	-	87.7
100% OPC	0.35	0.487	normal	76.4
80% OPC + 20% HCFA	0.31	0.520	-	82.4
80% OPC + 20% HCFA	0.39	0.469	normal	72.0
80% OPC + 20% LFS	0.28	0.549	-	81.8
80% OPC + 20% LFS	0.35	0.497	normal	68.8
80% OPC + 20% LF	0.26	0.567	-	80.6
80% OPC + 20% LF	0.35	0.496	normal	68.1
70% OPC + 20% HCFA + 10% LFS	0.30	0.539	-	77.0
70% OPC + 20% HCFA + 10% LF	0.29	0.534	-	84.4

## Data Availability

The data presented in this study are available on request from the corresponding author.
